# Prediction of walking speed one year following hip fracture based on pre-fracture assessments of mobility and physical activity

**DOI:** 10.1186/s12877-024-04926-1

**Published:** 2024-04-22

**Authors:** Kristi Elisabeth Heiberg, Monica Beckmann, Vigdis Bruun-Olsen

**Affiliations:** 1 ¹Department of Rehabilitation Science and Health Technology, Faculty of Health Sciences, Oslo Metropolitan University, Oslo, Norway; 2 ²Department of Medical Research, Clinic of Bærum Hospital, Vestre Viken Hospital Trust, Drammen, Norway

**Keywords:** Hip fracture, Walking, Physical activity, Predictors, Recalled assessments

## Abstract

**Background:**

Older people with hip fracture are often medically frail, and many do not regain their walking ability and level of physical activity. The aim of this study was to examine the relationship between pre-fracture recalled mobility, fear of falling, physical activity, walking habits and walking speed one year after hip fracture.

**Methods:**

The study had a longitudinal design. Measurements were performed 3–5 days postoperatively (baseline) and at one year after the hip fracture. The measurements at baseline were all subjective outcome measures recalled from pre-fracture: The New Mobility Scale (NMS), the ‘Walking Habits’ questionnaire, The University of California, Los Angeles (UCLA) Activity Scale, Fear of Falling International (FES-I) and demographic variables. At one year 4-meter walking speed, which was a part of the Short Physical Performance Battery (SPPB) was assessed.

**Results:**

At baseline 207 participants were included and 151 were assessed after one year. Their age was mean (SD) 82.7 (8.3) years (range 65–99 years). Those with the fastest walking speed at one year had a pre-fracture habit of regular walks with a duration of ≥ 30 min and/or a frequency of regular walks of 5–7 days a week. Age (*p* =.020), number of comorbidities (*p* <.001), recalled NMS (*p* <.001), and recalled UCLA Activity Scale (*p* =.007) were identified as predictors of walking speed at one year. The total model explained 54% of the variance in walking speed.

**Conclusions:**

Duration and frequency of regular walks before the hip fracture play a role in walking speed recovery one year following the fracture. Subjective outcome measures of mobility and physical activity, recalled from pre-fracture can predict walking speed at one year. They are gentle on the old and medically frail patients in the acute phase after hip fracture, as well as clinically less time consuming.

## Background

Hip fracture is a common consequence of falls in older people and may have severe consequences for the individual and for society [1, 2]. A broken hip is to many experienced as a disruptive and major life event that is potentially life-changing [3, 4]. Long after the fracture the patients often have disabilities [5] and significantly less independence in physical activities, especially walking [1].

Walking speed is the result of a complex interplay of body structures and functions, proactive and reactive postural control [6], lower extremity strength [7], aerobic capacity [8], and vision [9]. Furthermore, it is a valid, reliable, and sensitive measure of functional status and overall health in a wide range of populations, and walking speed has been designated as the ‘6th vital sign’ of function [10]. From prior research it is known that there is a consistent association between walking speed and mortality [11]. Furthermore, an association has been found between walking difficulty and physical activity among older adults. Those with walking difficulties have lower physical activity when compared to those without [12]. Therefore, improving mobility outcomes, such as walking speed, is key in recovery after hip fracture [13].

There are discrepancies regarding the recovery trajectory of walking and mobility following hip fracture. In one study, most of the recovery of walking ability and activity of daily living occurred within six months after the fracture [1], while another study reported that it may take up till nine months to plateau walking ability, balance, and muscular strength [14]. From prior studies it’s well known that patients’ functioning in physical and social domains do not return to pre-fracture levels within the first six months [15]. Only 50% of the patients had recovered their walking ability at six months [16]. Additionally, several studies have indicated that patients often have worse mobility than age-matched controls 1–2 years following hip fracture [17–19]. Tang et al. (2016) showed that among those with a high pre-fracture physical functioning, such as the ability to walk a block or climb one flight of stairs, only approximately 30% returned to their prior level of physical functioning [20]. These findings supported that many patients with hip fracture never regain their pre-fracture walking ability. Moreover, the patients with hip fracture often have medical frailty, which is connected to several comorbidities, resulting in vulnerability and reduced ability to recover from a stressor event. De Munter et al. (2022) found that frailty was associated with poor recovery, including walking ability [21].

Some studies examining predictive factors for long-term outcomes of physical functioning have applied objective outcome measures of walking speed, such as the Timed Up & Go (TUG) test [22] and the Short Physical Performance Battery (SPPB) test, with the 4 m walking included [23]. The assessments were performed a few days after surgery. Gherardini et al. (2019) examined whether 4-m walking speed measured in the acute phase before discharge from hospital could predict functional recovery one year after hip fracture surgery. They concluded that walking speed could predict long-term functional changes and clinical outcomes, such as institutionalization and death [23]. However, such objective performance-based outcome measures in the early acute phase may be demanding for the older patients who also may have medical frailty [21, 24]. Our experience is that some are unable to perform the tests. In the HIPFRAC trail, which this study was a part of 20% of the participants were unable to perform SPPB walking 4 m in the acute phase before hospital discharge. We have not found any studies in which only subjective outcome measures of mobility and physical activity recalled from pre-fracture were applied to predict walking speed one year after hip fracture.

Presently, our hypothesis is that pre-fracture recalled subjective measures of mobility, fear of falling, and physical activity, including walking habits have a predictive value of long-term walking speed after hip fracture. The aim of this study was to examine the relationship between mobility, fear of falling, physical activity, and walking habits recalled from pre-fracture and walking speed one year after hip fracture.

## Methods

This longitudinal study with older participants with hip fracture is part of a larger study called the HIPFRAC trial, which included a prospective longitudinal study [25] and a randomised controlled trial (RCT) [26]. The RCT examined the effect of additional exercises and usual physiotherapy compared with usual physiotherapy alone. No differences between the groups were found [26]. The participants with hip fracture were admitted to a hospital in Norway for surgery during the period from May 2016 to March 2019. They were eligible if they were ≥ 65 years, living at home before the low-energy hip fracture, able to walk 10 m with or without a walking aid, and able to understand the Norwegian language. If they had a pathological fracture or a multi-trauma injury, had less than 3-month life expectancy, or had severe cognitive impairments, such as inability to answer questions adequately and understand instructions during exercise and assessments they were excluded from participation [27].

Two physiotherapists recruited all the participants while they were hospitalized and performed all outcome measures. Measures were taken at baseline, i.e. before hospital discharge (3–5 days after surgery) and one year after surgery.

The trial was approved by the Regional Committee for Ethics in Medical Research (South-East Norway) (2015/2147). All participants gave their informed written consent, and the study was conducted according to the World Medial Association Declaration of Helsinki.

## Outcome measures

### Walking speed measured one year after surgery (dependent variable)

*Walking speed* was measured by a 4-m walking test, which was a part of the Short Physical Performance Battery (SPPB). The participants were instructed to walk in their usual comfortable speed on flat floor with or without a walking aid and time was measured in seconds [28]. Walking speed (m/sec) was calculated from the number of seconds used to walk 4 m. The SPPB is a valid and reliable measurement when used in older people [28, 29].

### Self-reported measures at baseline, recalled from pre-fracture (independent variables)

*The ‘Walking habits’ questionnaire* measures physical activity [30, 31]. The questionnaire reports the duration and frequency of the participants’ regular walks before the fracture. The questions used were ‘Do you take a daily walk?’ and ‘For how long do your walks generally last?’ These questions are scored on 6-point and 5-point scales [30]. The questionnaire is a valid measurement for walking habits and physical activity in older people [30]. Walking habits were recorded at baseline (recalled from the last three- to four weeks before the fracture).

*The University of California, Los Angeles (UCLA) Activity Scale* is a questionnaire that measures the participants’ level of physical activity [32]. Level of physical activity is evaluated on a 10-point scale based on 10 descriptive activity levels ranging from wholly inactive and dependent (level 1) to regular participation in impact sports (level 10). In the questionnaire the participants are asked about their participation in the various activities, and the researcher rates the categories. The UCLA Activity Scale has been found reliable, valid, and adequate in persons with total hip arthroplasty [32, 33]. We used a Norwegian version translated from English according to a standard procedure [34]. This version was not tested for reliability and validity. It was recorded at baseline (recalled from the last three- to four weeks before the fracture).

Fear of falling was measured by the *Fall Efficacy Scale International (FES-I) questionnaire* [35, 36]. Sixteen questions are scored on a 4-point scale ranging from ‘not at all concerned’ (16 points) to ‘very concerned about falling’ (64 points). FES-I was recorded at baseline (recalled from the last three- to four weeks pre-fracture).

New Mobility Scale (NMS) measures the patients ability to perform indoor- and outdoor walking and shopping during the last few weeks [37]. The score provided is a 0–3 score for each function where 0 = no walking ability, 1 = with help from another person, 2 = with an aid, and 3 = no difficulty and no aid, resulting in a total score from 0 (no walking ability at all) to 9 (fully independent) [38]. Physical function is classified as either low (0–6) or high (7-9) [37]. NMS was recorded at baseline (recalled from pre-fracture).

*Demographic variables*, such as age, sex, body mass index (BMI), educational level, marital status, comorbidities, assistance from family members, nursing at home, previous falls indoors and outdoors, use of walking aid indoors and outdoors, and type of fracture and surgery were collected at baseline.

### Statistical analysis

Descriptive data are presented as means and with 95% confidence intervals (CI) or in numbers and percentages. The continuous data were mainly normal distributed. The differences between dropouts and those who fulfilled at one year were analyzed by independent sample *t* tests.

Walking speed at one year was the dependent variable. This was part of the SPPB. Associations between walking speed at one year and the independent variables (age, sex, BMI, education level, cohabiting (y/n), number of comorbidities, comorbidities (y/n), frequency of regular outdoor walks, duration of regular outdoor walks, use of a walking aid indoors and outdoors, NMS, UCLA Activity Scale, and FES-I) were analyzed by Pearson’s correlation analyses. Variables that fulfilled the correlation criteria (*p* <.05) were included in the multiple linear regression analysis. The predictors with the smallest contribution to explain the variance of walking speed at one year were excluded from the model by manual backward stepwise procedure. The best subset of statistically significant predictors was selected. In the end the scatter plots of distribution of the residuals for the models were checked and found acceptable. The regression coefficient is reported with 95% CI. *P* values of < 0.05 were considered statistically significant. All analyses were conducted with SPSS statistical software version 25 (IBM Corp).

## Results

**Fig. 1:**
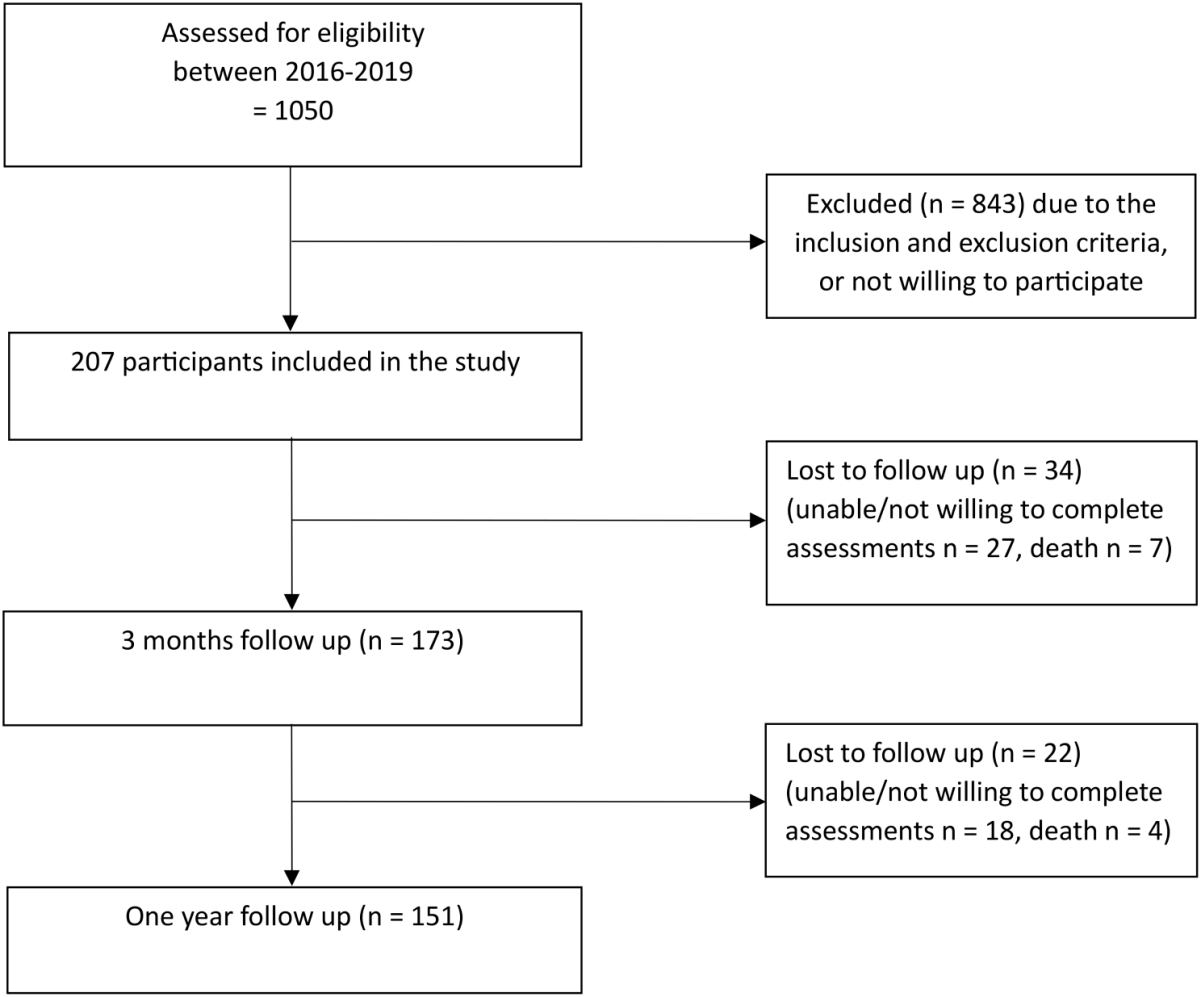
Cohort diagram of the study

We included 207 participants at baseline. Their age was mean (SD) 82.7 (8.3) years (range 65–99 years), and 76.8% were women. Reported comorbidities were mean (SD) 1.48 (1.3) (Table 1). Their most common comorbidities were osteoarthritis (32%), osteoporosis (30%), heart disease (25%), cancer (20%), stroke/hemiplegia (15%), and lung disease (10%). At one year, 45 participants withdrew from the follow-up assessments and 11 participants had died (Fig. 1). At baseline, the 56 participants who dropped out at one year were statistically significant four years older, had lower ability in walking and shopping (-1 point in NMS), were less physically active (-1.3 points in UCLA Activity Scale), and had more fear of falling (+ 6.5 points in FES-I) than those (*n* = 151) who completed the study (*p* <.001). There were no statistically significant differences in BMI and number of comorbidities (*p* >.05).

**Table 1:** Characteristics of the participants with hip fracture at baseline (recalled from pre-fracture) in the total cohort (*n* = 207) and the maintaining cohort at one year (*n* = 151)

Characteristics	Total cohort (*n* = 207)		Cohort fulfilled at one year (*n* = 151)	
	n (%)	Mean (SD)	n (%)	Mean (SD)
Age (y)		82.7 (8.3)		81.5 (8.1)
Women	159 (76.8)		118 (78.1)	
Body Mass Index (BMI)		23.5 (8.5)		23.0 (3.6)
Educational level ≤ 12 y	122 (58.9)		88 (58.2)	
Living alone	115 (55.6)		79 (52.3)	
Comorbidities (number)		1.5 (1.3)		1.4 (1.3)
Nursing at home (yes) (*n* = 204)	52 (25.0)		30 (20.0)	
Assistance from family member (yes) (*n* = 201)	58 (27.9)		33 (21.9)	
Previous falls (yes) (last 6 months)	85 (41.1)		60 (39.7)	
Previous falls indoors (last 6 months)	128 (61.8)		44 (29.1)	
Previous falls outdoors (last 6 months)	74 (35.7)		29 (19.2)	
Use of walking aid indoor	69 (33.3)		38 (25.2)	
Use of walking aid outdoors	101 (48.8)		68 (45.0)	
Type of hip fracture (surgery treatment) (*n* = 206) (*n* = 150)				
Fractura colli femoris (two parallel screws)	25 (12.1)		15 (9.9)	
Fractura colli femoris (hemiarthroplasty)	113 (54.6)		87 (57.6)	
Per trochanteric fracture (dynamic hip screw)	59 (28.5)		41 (27.2)	
Sub trochanteric fracture (intramedullary hip screw)	8 (3.9)		7 (4.6)	

**Fig. 2:**
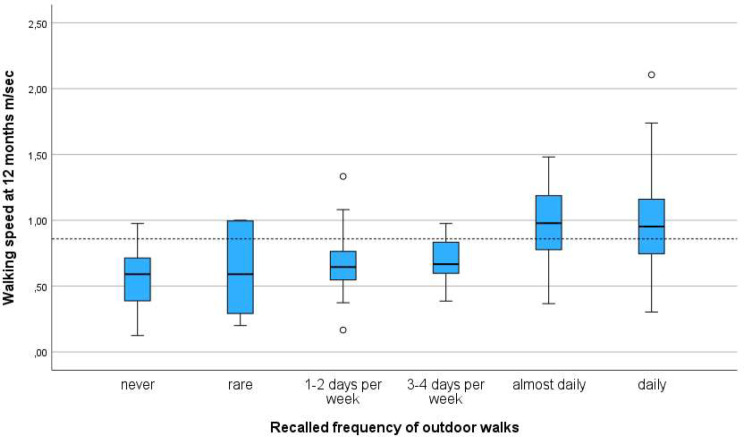
Distribution of walking speed one year following hip fracture and frequency of outdoor walks recalled from pre-fracture

**Fig. 3:**
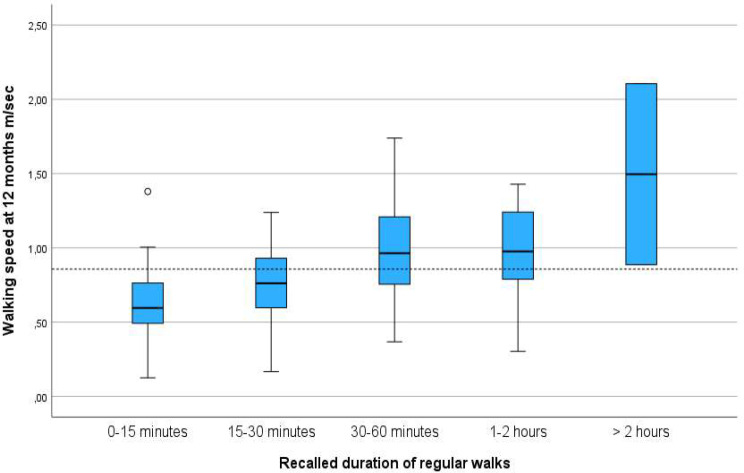
Distribution of walking speed one year following hip fracture and duration of regular outdoor walks recalled from pre-fracture

The participants with the fastest walking speed after one year were the 124 who performed regular outdoor walks 5–7 days a week pre-fracture (Table 2) (Fig. 2) and the 97 participants who performed regular outdoor walks of ≥ 30 min (Table 2) (Fig. 3).

**Table 2:** Outcomes of mobility and physical activity at baseline recalled from pre-fracture in participants with hip fracture

Instruments	Total cohort ^a^ (*n* = 207)		Cohort fulfilled at one year ^b^ (*n* = 151)	
	n (%)	Mean (SD)	n (%)	Mean (SD)
*‘Walking habits’*				
Duration of regular walks (*n* = 187^a^, 149^b^)				
0–15 min	29 (14.0)		26 (17.2)	
15–30 min	61 (29.5)		46 (30.5)	
30–60 min	71 (34.3)		57 (31.7)	
60–120 min	23 (11.1)		20 (13.2)	
120 min	3 (1.4)		2 (1.3)	
Frequency of regular walks (*n* = 204^a^, 150^b^)				
Never	14 (6.8)		8 (5.3)	
Almost never	18 (8.7)		6 (4.0)	
1–2 times a week	25 (12.1)		19 (12.6)	
3–4 times a week	23 (11.1)		18 (11.9)	
Almost every day	25 (12.1)		24 (15.9)	
Daily walks	99 (47.8)		75 (49.7)	
*NMS total score* (score 0–9) (*n* = 204^a^, 149^b^)		7.2 (2.0)		7.5 (1.9)
Indoor walking (score 0–3) No difficulty and no aid	137 (66.2)	2.7 (0.5)	112 (74.2)	2.8 (0.4)
With a walking aid	66 (31.9)		37 (24.6)	
With help from another person	1 (0.5)		0 (0)	
Not at all	0 (0)		0 (0)	
Outdoor walking (score 0–3)		2.4 (0.7)		2.5 (0.6)
No difficulty and no aid	104 (50.2)		85 (56.3)	
With a waling aid	87 (42.0)		59 (39.1)	
With help from another person	8 (3.9)		2 (1.3)	
Not at all	5 (2.4)		3 (2.0)	
Walking during shopping		2.1 (1.0)		2.2 (1.0)
No difficulty and no aid	101 (48.8)		84 (55.6)	
With a walking aid	47 (22.7)		30 (19.9)	
With help from another person	34 (16.4)		20 (13.2)	
Not at all	22 (10.6)		15 (9.9)	
*UCLA Activity Scale* (score 0–10) (*n* = 204^a^, 149^b^)		4.6 (1.9)		5.0 (2.0)
*FES-I* (score 16–64) (*n* = 201^a^, 146^b^)		25.8 (10.2)		24.0 (9.2)

NMS = New Mobility Score, UCLA Activity Scale = University of California, Los Angeles Activity Scale, FES-I = Fall Efficacy Scale International

The participants reported in NMS a score of mean (SD) 7.2 (2.0) which indicated a high level of physical function. UCLA Activity Scale showed mean (SD) 4.6 (1.9) points and indicated participation in low to moderate activities. Further, their score on FES-I was mean (SD) 25.8 (10.2) points and indicated low concern about falling (Table 2).

In the correlation analyses the pre-fracture variables that correlated significantly with walking speed at one year (*p* <.05) were age (*r* = −.518), education (*r* =.207), marital status (*r* =.272), number of comorbidities (*r* = −.373), nursing at home (*r* = −.285), assistance from family member (*r* = −.271), frequency of walks outdoors (*r* =.464), duration of regular walks outdoors (*r* =.388), whether they used a walking aid while walking indoors (*r* =.529) and outdoors (*r* =.590), NMS total score (*r* =.645), UCLA Activity Scale (*r* =.646), and FES-I (*r* = −.509). These variables fulfilled the correlation criteria and were included in the multiple regression analysis, together with sex (*r* = −.006) and BMI (*r* = −.009) (*p* >.05).

There were 151 participants that completed the 4-m walking speed test, and these were included in the multiple regression analysis. Their walking speed at one year was mean (SD) 0.86 m/sec. (0.34) (range 0.13 m/sec– 2.11 m/sec).

Multiple linear regression analysis showed that older age (*p* =.020), number of comorbidities (*p* <.001), recalled NMS (*p* <.001), and recalled UCLA Activity Scale (*p* =.007) were identified as predictors of walking speed one year after hip fracture. The total model explained 54% of the variance in walking speed (Table 3).

**Table 3:** Predictors of walking speed one year following hip fracture from recalled self-reported measures (*n* = 151)

Variables		Crude Estimates				Adjusted Estimates		
	B	Beta	95% CI	P	B	Beta	95% CI	P
*Walking speed (m/sec) at 12 months*								
Age (y)	− 0.007	− 0.171	[-0.014, 0.000]	0.042	− 0.007	− 0.169	[-0.013, − 0.001]	0.020
Sex (ref. male)	− 0.048	− 0.061	[-0.149, 0.052]	0.344				
BMI	0.001	− 0.007	[-0.012, 0.011]	0.909				
Education (y) (range < 7 - ≥17)	0.021	0.085	[-0.010, 0.051]	0.179				
Married/cohabiting	0.017	0.028	[-0.064, 0.098]	0.684				
Number of comorbidities	− 0.060	− 0.237	[-0.092, − 0.028]	< 0.001	− 0.055	− 0.216	[-0.084, − 0.025]	< 0.001
*Recalled self-reported variables*								
Nursing at home	− 0.001	− 0.005	[-0.032, 0.029]	0.943				
Assistance from family member	− 0.006	− 0.028	[-0.036, 0.024]	0.689				
‘Walking Habits’ question “frequency of outdoor walks”	0.008	0.038	[-0.028, 0.043]	0.668				
‘Walking Habits’ question “duration of regular walks”	0.004	0.011	[-0.053, 0.060]	0.900				
Use of a walking aid outdoors	0.116	0.174	[0.027, 0.260]	0.110				
Use of a walking aid indoor	− 0.072	− 0.102	[-0.228, 0.083]	0.357				
NMS	0.035	0.208	[-0.019, 0.089]	0.207	0.054	0.326	[0.026, 0.083]	< 0.001
UCLA Activity Scale	0.032	0.186	[-0.004, 0.067]	0.084	0.042	0.246	[0.011, 0.072]	0.007
FES-I	− 0.002	− 0.047	[-0.007, 0.004]	0.602				

*Note* Unstandardized B, Standardized Coefficients Beta, 95% CI, and *P* value given for crude estimates and adjusted estimates in the multiple regression analyses. The total model explained 54% of the variance in walking speed at 12 months (*R*^*2*^= 0.537)

BMI = Body Mass Index. NMS = New Mobility Score. UCLA Activity Scale = University of California, Los Angeles Activity Scale. FES-I = Fall Efficacy Scale International

## Discussion

In this study, the duration and frequency of pre-fracture walks were associated with the fastest walking speed at one year after hip fracture. The subjective recalled outcome measures of NMS and UCLA Activity Scale, together with age and number of comorbidities were identified as predictors of walking speed one year following hip fracture. The variables explained 54% of the variance in walking speed. To our knowledge, outcome measures recalled from pre-fracture are not previously used to predict long-term level of physical activity and -function in older people following a hip fracture.

It is well known that physical activity is important in older age to prevent a decline in independence and physical function [39]. There is a significant association between pre-fracture health status and being alive five years following a hip fracture [40]. Although the benefits of regular physical activity are well known, majority of older people do not meet the minimum physical activity levels recommended by the World Health Organization (WHO) [41]. In the WHO guidelines on physical activity from 2020 it is recommended that adults undertake at least 150 min per week of moderate-intensity physical activity [42]. It seemed that our participants who were living at home before the fracture, were physically active according to our data. They scored 4.6 points on UCLA Activity Scale, which indicated that they participated regularly in mild to moderate activities, such as housework, shopping, and swimming. Additionally, 60% reported that they went for nearly daily walks and nearly 50% reported a duration of their walks of more than 30 min. One year following hip fracture the average walking speed was above the cut-off value of 0.8 m/sec, indicating that the participants were independent in self-care, able to do housework activities, and able to ambulate in the community [10].

Prior research has found that hip fractures usually occur in older people that have medical problems or comorbidities [43]. In the multiple regression analysis, we identified older age and the number of comorbidities as predictors of walking speed one year after the fracture. This is in line with the findings in a systematic review by Xu et al. (2019). Older age and the presence of comorbidities predicted poor functional outcomes [44]. They also stated that an adequate treatment of certain potentially modifiable predictors could improve the prognosis of some patients.

Our model explained 54% of the variance in walking speed at one year. Several other plausible aspects may play an additional role. Especially the participants’ walking speed before the fracture would have been an interesting variable, but after acute incidences we do not have pre-fracture objective measures of physical functioning.

Identification of those at risk of a poor outcome in walking after hip fracture would prove helpful in rehabilitation planning after hip fracture [23]. Easily applicable pre-fracture recalled outcome measures in the acute phase would enable clinicians to detect those patients who are likely to have a poor outcome of physical function in the long run, and thereby support them at an earlier stage to improve their future recovery. The use of such measures would be gentler on the patients and more resource-saving for the clinicians during a busy working day.

## Strengths and limitations

In this study we chose reliable and validated measurements often used after hip fracture. For example, European Society for Clinical and Economic Aspects of Osteoporosis, Osteoarthritis and Musculoskeletal Diseases (ESCEO) have recommended the 4-m walk speed to measure physical performance in older people [45], and assessments of short walking distances are the most frequent in clinical studies [46]. The measurements were simple and not time-consuming, only requiring easily applicable recalled measurements of mobility, activity level and fear of falling. The number of participants was relatively high and with different ages and a variety of functional status. Furthermore, the participants comprised of both those who went directly home and those who needed a short-term stay at a nursing home after discharge from hospital, making our results representative for older persons with hip fracture in Norway. Taken together, these inclusion criteria strengthen external validity.

There were some limitations in the study. Those participants with severe cognitive impairment were excluded from participation. This may limit the generalizability of the study. We did not collect specific data on cognitive function. However, the assessor evaluated whether the patients were able to understand instruction in the assessment- or exercise situation.

Recalled walking ability is a complex cognitive task, and some participants may have over- or under-reported their pre-fracture walking ability [47, 48]. Therefore, we could additionally have asked their family or caregivers. However, we experienced that the participants seemingly had few difficulties in remembering their walking habits, level of physical function, mobility, physical activity, and fear of falling from before the fracture when they were asked 3–5 days postoperatively. Nevertheless, there is a risk of recalled bias regarding these assessments. The participants were recruited from only one hospital and two municipalities in a part of Norway in which the population has a high educational level. In future studies, to strengthen the results the participant sample should be drawn from different hospitals in different parts of the country, and from hospitals abroad.

## Conclusions

Our findings supported that there is a predictive validity in subjective recall on functional walking and 1-year post-operative walking speed. The participants’ physical function, level of physical activity, together with older age and number of comorbidities were identified as predictors of walking speed one year following a hip fracture. These predictors explained more than half the variance in walking speed. Duration and frequency of pre-fracture regular walks played a role in walking speed after one year. Recalled subjective outcome measures are less demanding than performance-based measures in the acute postoperative phase after hip fracture. They are resource-saving and have a potential to early identify patients with a possible risk of poor walking outcome.

## Data Availability

The datasets generated and analysed during the current study are not publicly available due to the limited permission given from the Regional Committee for Ethics in Medial Research (South-East Norway) but are available from the corresponding author on reasonable request.
